# Medication-related osteonecrosis of the jaw using periodontitis-induced rat before tooth extraction

**DOI:** 10.1186/s12903-023-03200-x

**Published:** 2023-08-12

**Authors:** Kyeong-Mee Park, Jieun Cheong, Nan-Sim Pang, Kee-Deog Kim, Jung-Seok Lee, Wonse Park

**Affiliations:** 1 Department of Advanced General Dentistry and Human Identification Research Institute, Yonsei University College of Dentistry, 50-1 Yonsei-ro, Seodaemun-gu, Seoul, 03722 South Korea; 2 Department of Advanced General Dentistry, Yonsei University College of Dentistry, 50-1 Yonsei-ro, Seodaemun-gu, Seoul, 03722 South Korea; 3 Department of Periodontology, Research Institute for Periodontal Regeneration, Yonsei University College of Dentistry, 50-1 Yonsei-ro, Seodaemun-gu, Seoul, 03722 South Korea

**Keywords:** MRONJ, Bisphosphonate, Zoledronic acid, Periodontitis, Tooth extraction, Ovariectomy, Rat

## Abstract

**Objective:**

This study aimed to investigate the occurrence of medication-related osteonecrosis of the jaw (MRONJ) after tooth extraction due to periodontitis in ovariectomized rats.

**Methods:**

Twenty-four osteoporosis-induced rats were administered with zoledronic acid (ZA; ZA group) or saline (CONT group). In both groups, tooth extraction was performed after inducing periodontitis, and all animals were sacrificed 8-week after tooth extraction.

**Results:**

Micro-CT of the tibia showed that the bone volume fraction, bone surface density, trabecular number, and bone mineral density were significantly higher in the ZA group than in the CONT group. Histologically, the proliferative zone on the growth plate was thicker in the ZA group than in the CONT group. Micro-CT of the extraction sites revealed that the bone volume fraction was significantly higher in the ZA group than in the CONT group. Radiologically, the ZA group showed partial healing and delayed healing. Histological analysis revealed normal bone healing status with completely healed epithelium in the extraction sites of the CONT group, whereas abnormal empty osteocytes in the necrotic bone and inflammatory infiltration were observed in the ZA group.

**Conclusion:**

The incidence of MRONJ increased in the rats administered with ZA.

## Introduction

Bisphosphonates (BP) are representative drugs affecting bone metabolism and are widely used in osteoporosis treatment. They prevent bone resorption by inhibiting the activation of osteoclasts [1–3]. BP are also used to treat bone diseases in multiple myeloma and bone metastases associated with solid tumors by inhibiting the bone resorption and angiogenesis [4–6]. The most serious side effect of BP is osteonecrosis of the jaw (ONJ). ONJ was first reported after dental treatment in BP-treated patients by Marx in 2003 [7]. There have been many reports of side effects associated with BP since Marx’s report, and BP-associated jaw necrosis was defined as bisphosphonate-related osteonecrosis of the jaw (BRONJ) [8]. Recently, it has been defined as medication-related osteonecrosis of the jaw (MRONJ) in association with anti-resorptive or anti-angiogenic medications, such as denosumab [9, 10].

The incidence of MRONJ is reportedly related to the duration, type, and dosage of BP administration [11, 12]. Particularly, higher incidence of MRONJ has been reported with high-dose intravenous zoledronic acid (ZA) than with oral BP [8, 13]. Dental causes considered as risk factors for MRONJ include tooth extractions following periodontal or periapical diseases and dental surgeries, such as periodontal flap surgery or micro-endodontic surgery [14, 15]. The major manifestation of MRONJ is exposure of the alveolar bone, which is accompanied by pain, edema, paresthesia, suppuration, and soft tissue ulceration [15]. The most serious problem with MRONJ is that effective treatment is evasive, despite the best oral healthcare practices including antibiotic therapy, parathyroid hormone therapy, and surgical debridement/resection [9, 16, 17]. Because the treatment of MRONJ is usually difficult and recurrence is frequent, efficient prevention of MRONJ in susceptible patients is vital.

The pathophysiology of MRONJ has not been elucidated completely, and several hypotheses have been suggested to explain its mechanism [18–21]. Many previous studies have used animal models that differ from the actual patients in clinic [22–24]. Most patients with MRONJ are female, old, and menopausal [8]. They have local inflammatory conditions such as periodontal or periapical diseases and general immunosuppressive conditions [25–27]. It has been reported that removal of inflammation prior to tooth extraction reduces the incidence of osteonecrosis [28]. Kim et al. emphasized the importance of dental pulp and periodontal disease as local factors in the development of MRONJ [28]. However, in the case of animal experiments, healthy teeth are extracted, which is a model that has difficulty in understanding the mechanism of MRONJ. Therefore, it is important to develop an animal model similar to the situations of such patients, with the local and systemic risk factors for MRONJ, in order to perform animal experiments that can be applied in clinics.

In this study, the effect of periodontitis on the pathogenesis of MRONJ was investigated using a periodontitis-induced animal model before tooth extraction.

## Methods

### Animals

In this study, 24 skeletally mature, 12-week-old, female Sprague-Dawley rats (Orientbio Co., Ltd.; Seongnam-si, Gyenggi-do, Korea) were used. The mean weight of the animals was 281 g at 12 weeks of age and 421 g immediately before sacrifice. The animals were housed under standard laboratory conditions (temperature, 20 °C ± 5 °C; humidity, 50% ± 10%; lighting cycle, 12 h light/12 h dark), with 2–3 rats per cage and marked individually. All animals had *ad libitum* access to standard laboratory pellet diet and water. This investigation was carried out in accordance with the revised animals Act 1986 in the UK used for scientific procedure, and confirm the study complied with the ARRIVE guidelines. Animal selection, management, surgical protocol, and preparation followed a routine protocol approved by the Institutional Animal Care and Use Committee of Yonsei University Health System (IACUC No. 2016 − 0171).

### Experimental design

**Fig. 1:**
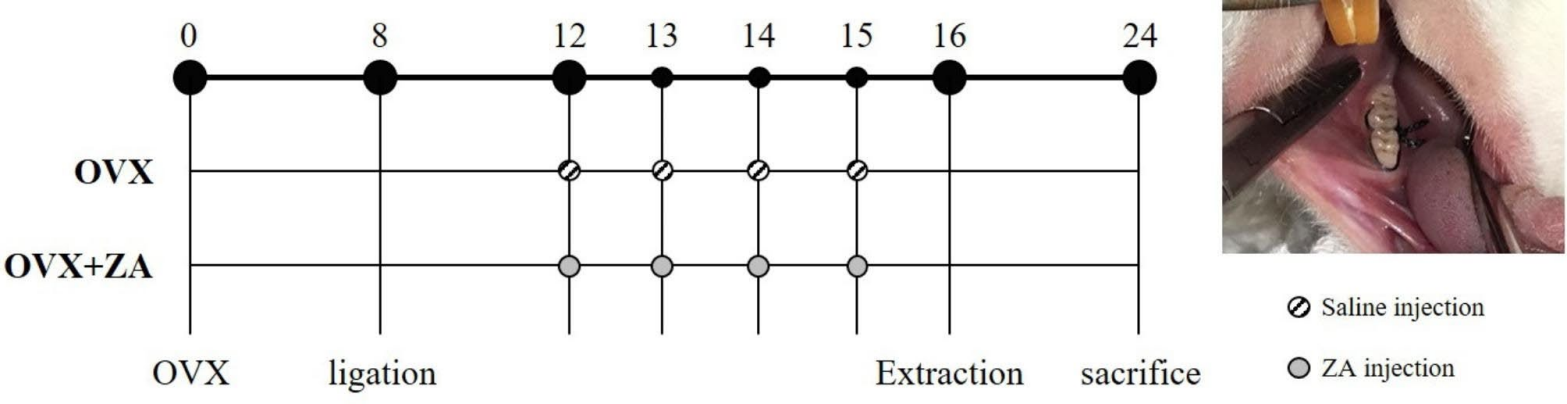
Experimental design protocol and clinical photographs of tooth ligation for inducing periodontitis

These 24 animals were randomly divided into two groups administered with saline (CONT group, N = 13) and zoledronic acid (ZA) (ZA group, N = 11), respectively (Fig. 1). Based on the results of the pilot study, the sample size was calculated, and G-power 3.1 was used. This was set at an effect size of 0.5, significance of 0.05, and power of 90%. Considering the dropout rate of 20%, 13 animals per group were finally calculated. At 12 weeks of age, bilateral ovariectomy (OVX) was performed in all animals under general anesthesia by intraperitoneal combination injection of tiletamine and zolazepam (50 mg/mL, 0.6 mL/kg; Zoletil®, Virbac Lab. Carros, France) and xylazine (23.32 mg/mL, 0.4 mL/kg; Rompun®, Bayer, Leverkusen, Germany). The OVX procedure is as follows: After shaving the spine, such as the lower abdomen, disinfect it. Afterwards, a 15 mm incision is made in the skin and a 5 mm incision is made in the peritoneum where the left/right ovary is located. The OVX is performed by exposing the ovary, and the remaining organs are placed back into the peritoneum. The peritoneum is not sutured, only the dorsal skin is sutured.

Postoperatively, meloxicam (1 mg/kg, once a day for 5 days; Metacam®, Boehringer Ingelheim, Rhein, Germany) and enfloxacin (10 mg/kg/day, once a day for 5 days; Baytril®, Bayer, Germany) were administered subcutaneously (SC).

Eight weeks later, 4 − 0 black silk ligation was performed on the cervical portion of the right mandibular first molar (M1) and second molar (M2) under general anesthesia in all rats (Fig. 1). Four weeks later, ZA (Zometa ready®, Novartis, Basel, Switzerland; 40 µg/kg, once a week for 4 weeks) was administered in the ZA group and saline (same volume as ZA) in the CONT group intravenously (IV) [29, 30].

Eight weeks later, silk remove with tooth extraction was performed under general anesthesia in all animals. Clinical observations such as gingival tenderness and tooth mobility around the ligated teeth were made before tooth extraction and silk removal. Local anesthesia was induced using 2% lidocaine (1: 80,000 epinephrine). The ligated right mandibular M1 and M2 were extracted using a sharp dental explorer after ensuring sufficient mobility. Bleeding was controlled by gauze pressure, and suturing was not required. After tooth extraction, meloxicam and enfloxacin were administered SC to prevent postoperative infection and achieve pain control.

All animals were sacrificed 8 weeks after tooth extraction by perfusion under general anesthesia. After removing the skin, the organs were extracted and fixed in 10% formalin.

### Micro-computed tomography (micro-CT)

Ten specimens (CONT group, N = 5; ZA group, N = 5) were subjected to micro-CT (Skyscan1173, Skyscan, Konitch, Belgium) at 100 kV, 100 µA, and 13.86 μm pixel size in the tibia and 18.12 μm pixel size in the jaw. CTAn (Skyscan, Aartselaar, Belgium) was used to reconstruct and analyze the images.

**Fig. 2:**
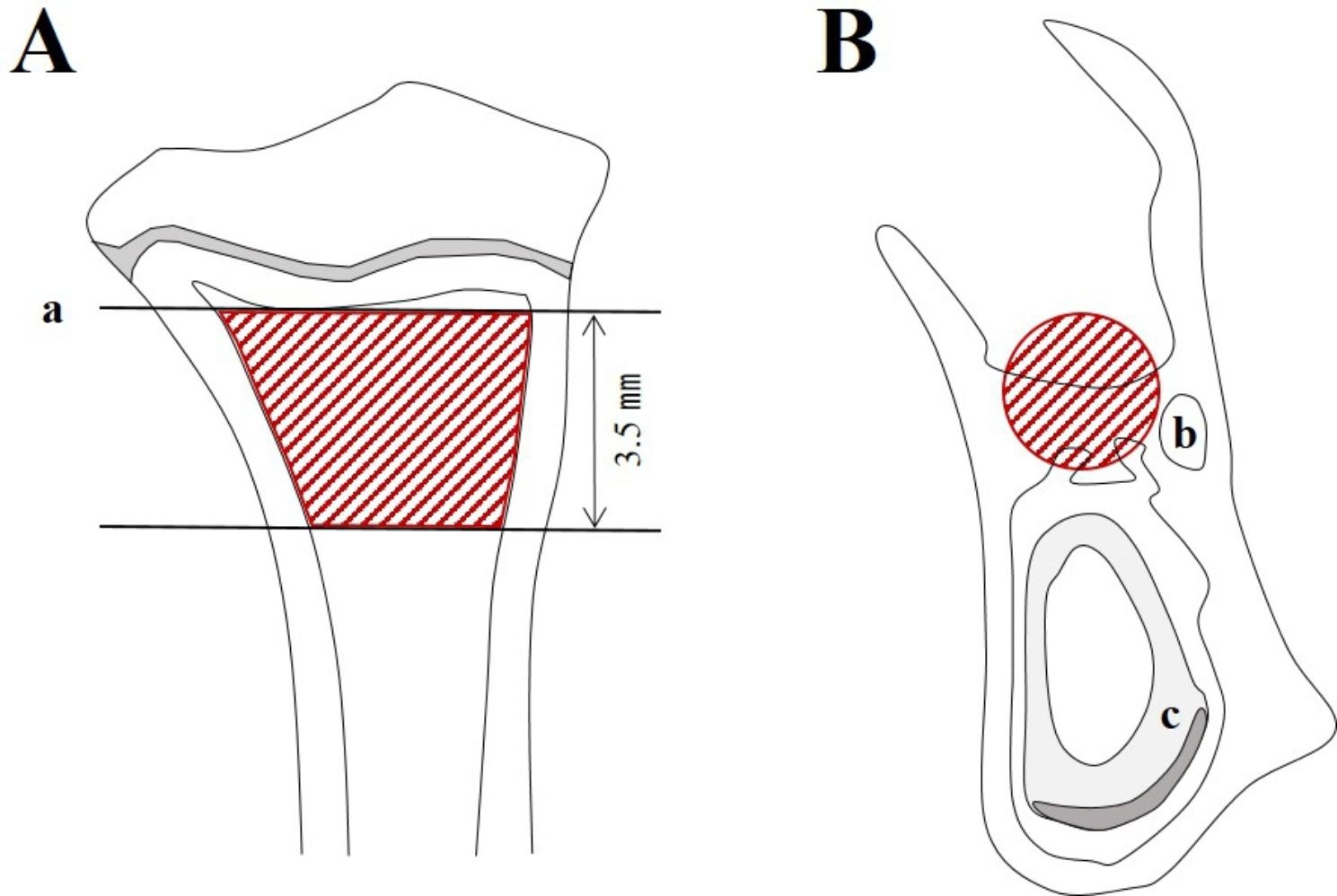
ROI in micro-CT analysis. **A** Proximal tibia, **B** EXT site. **a** Growth plate, **b** Mandibular canal, **c** Incisor root (ROI, red area) *Abbreviations*: ROI = region of interest, Micro-CT = micro-computed tomography, EXT = extraction

For the trabecular bone morphometry of the tibia, the region of interest (ROI) in the tibia was the growth plate and 3.5 mm of the upper trabecular bone (Fig. 2A and a) [31]. For the trabecular bone morphometry of the extraction site, the ROI was the interradicular bone of Ø0.6 mm-sized cylindrical shape in the coronal view (Fig. 2B, b and c). The posterior border was the mesial root surface of the third molar, and the anterior border was considered 4 mm from the posterior border.

The bone volume fraction (bone volume [BV]/total volume [TV], %), specific bone surface (bone surface [BS]/BV, mm^2^/mm^3^), bone surface density (BS/TV, mm^2^/mm^3^), trabecular number (Tb.N, 1/mm), trabecular thickness (Tb.Th, mm), trabecular separation (Tb.Sp, mm), and bone mineral density (BMD, g/cm^2^) of each specimen were assessed in trabecular bone morphometry [32].

### Histology

All specimens were decalcified using 10% ethylenediaminetetraacetic acid, embedded in paraffin, and 4-µm serial coronal sections were obtained. Hematoxylin and eosin staining was performed according to the manufacturer’s recommendations. Images were scanned using a light microscope (Case Viewer, 3DHISTECH Ltd., Budapest, Hungary). Histologically, MRONJ was diagnosed when one or more of the following signs were observed: exposed bone, necrotic bone, inflammatory infiltration, or sequestrum [33].

### Statistical analysis

Statistical analyses were performed using Statistical Product and Service Solutions (IBM SPSS 23.0, IBM Corp., Armonk, NY, USA). Mann-Whitney U-test and chi-squared test were used to compare parameters between the CONT and ZA groups; p-values < 0.05 were considered statistically significant.

## Results

Only 24 of 26 rats were included in the results. Two animals in the ZA group died. Both animals died of respiratory arrest during the tooth extraction process. The sample size was determined considering a dropout rate of 20%. Therefore, additional recruitment was not performed because the analyzed value of 24 rats did not affect the results.

No unexpected side effects (weight loss, behavioral abnormalities, serious infection at the experimental site) were found.

### Micro-computed tomography: proximal tibia

**Fig. 3:**
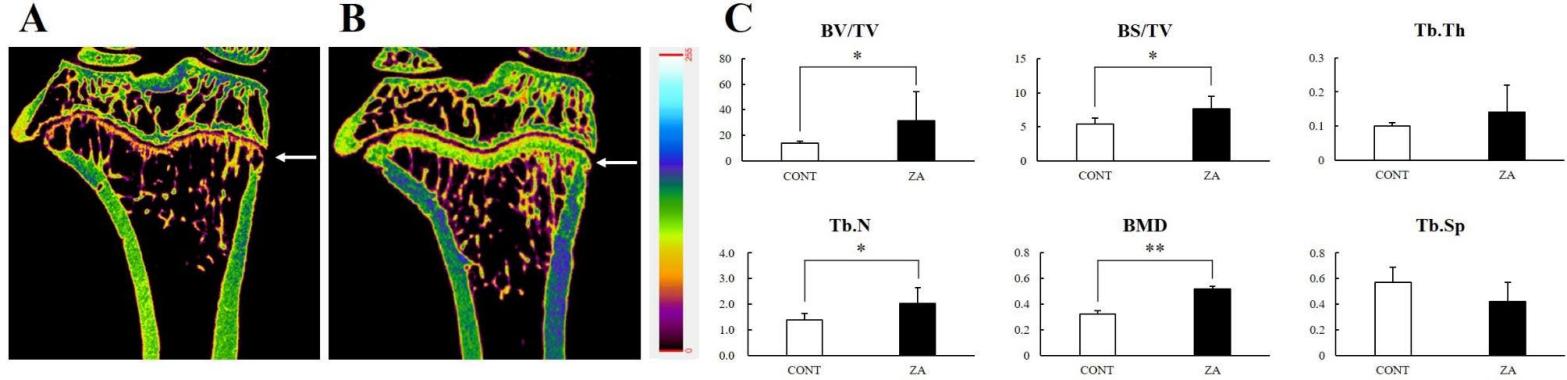
Treatment effect of ZA on proximal tibia. **A** Micro-CT image of the CONT group, **B** Micro-CT image of the ZA group, **C** Micro-CT analysis of trabecular bone (Growth plate, white arrow; *, *P* < 0.05; **, *P* < 0.01) *Abbreviations*: ZA = zoledronic acid, Micro-CT = micro-computed tomography

Figure 3a and b show the difference in the tibia between the two groups after sacrificing the rats. In the CONT group, a thin and low-density growth plate was observed, whereas thick and high-density growth plates and high-density trabecular patterns were observed in the ZA group. Furthermore, in the cortical bone of the tibia, the ZA group showed higher BMD than that of the CONT group. The BV/TV, BS/TV, Tb.N, and BMD values were significantly higher in the ZA group than in the CONT group (Fig. 3c).

### Micro-computed tomography: extraction socket

**Fig. 4:**
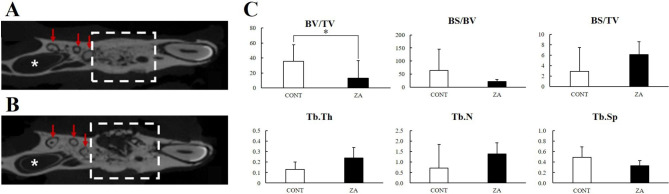
Treatment effect of ZA on the extraction site. **A** Micro-CT image of the CONT group, **B** Micro-CT image of the ZA group, **C** Micro-CT analysis of the trabecular bone (EXT socket, white square box; M3 root, red arrow; Incisor pulp chamber, white asterisk; *, *P* < 0.05) *Abbreviations*: ZA = zoledronic acid, Micro-CT = micro-computed tomography, EXT = extraction, M3 = 3rd molar

The extraction sockets showed different healing patterns between the ZA and CONT groups. The CONT group showed almost complete healing of the extraction socket, whereas the ZA group showed partial healing and delayed healing (Fig. 4a, b). The BV/TV of the extraction sockets was significantly higher in the CONT group than in the ZA group (Fig. 4c).

### Histology: proximal tibia

**Fig. 5:**
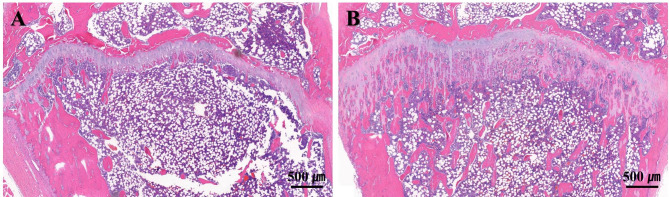
Longitudinal cross-sectional image of the proximal tibia (100 x magnification). **A** Growth plate region in CONT group, **B** Growth plate region in ZA group

As compared to the ZA group, the CONT group showed a thinner proliferative zone of the growth plate and less clear trabecular pattern (Fig. 5a). The ZA group showed a thicker proliferative zone of the growth plate than that of the CONT group, and many trabecular patterns were observed in the bone marrow of the former (Fig. 5b).

### Histology: extraction socket

**Fig. 6:**
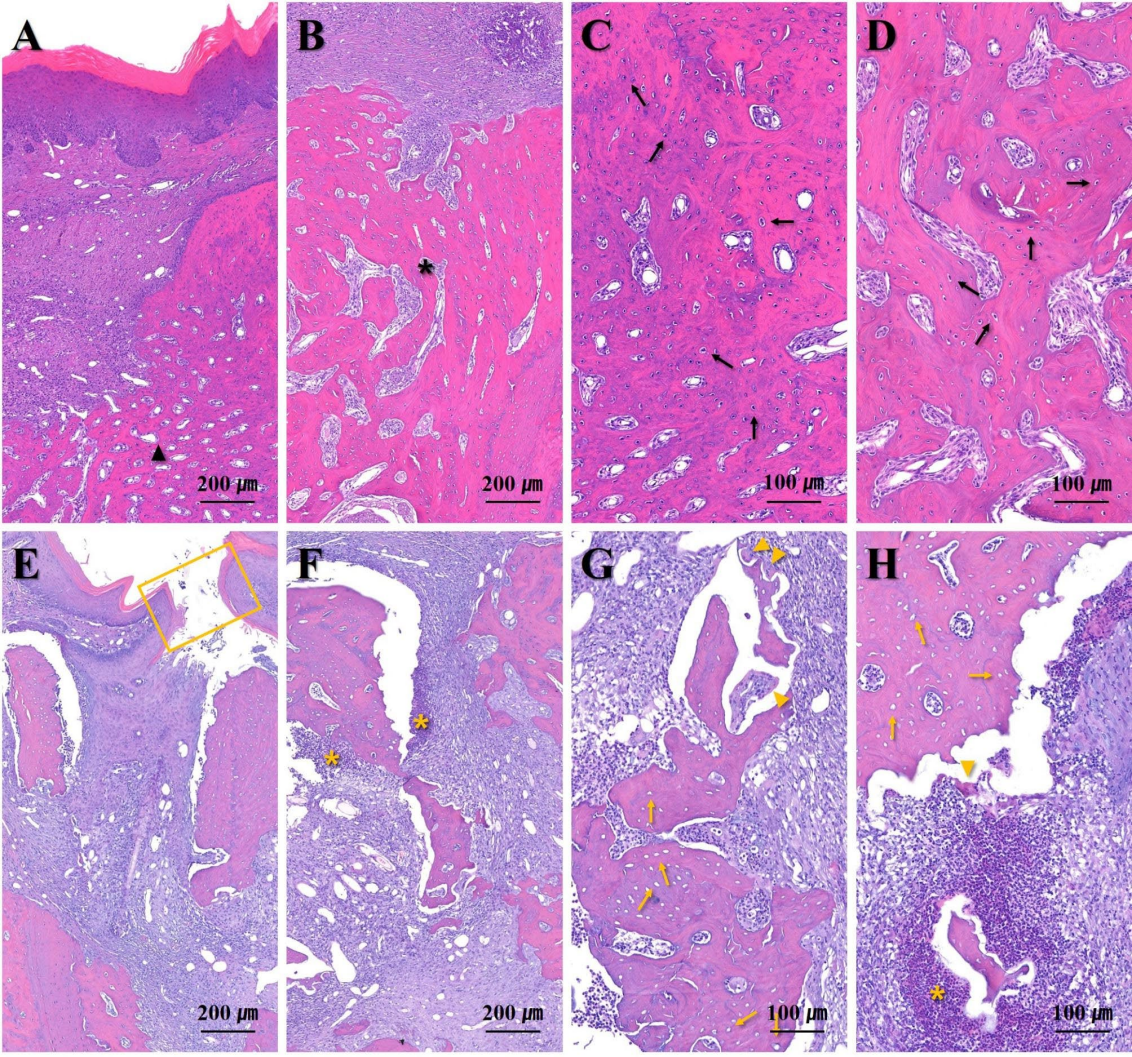
Representative images of the tooth extraction sites (100 and 200 x magnification). **A-D** CONT group, **E-H** ZA group (Normal bone healing state, black arrowhead; Completely filled EXT socket with bone, black asterisk; Normal osteocyte, black arrow; Opened wound, yellow box; Inflammatory infiltration, yellow asterisk; Empty lacunae, yellow arrow; Osteoclast, yellow arrowhead) *Abbreviations*: EXT = extraction

Normal bone healing with completely healed epithelium at the extraction sites was observed in the CONT group, as were many normal osteocytes (Fig. 6a-d). In contrast, abnormal empty osteocytes in the necrotic bone and inflammatory infiltration were observed in the extraction sockets of the ZA group (Fig. 6e-h). Also alveolar bone resorption and furcation involvement around the M1 and M2 areas were observed in all the rats.

### Incidence of MRONJ-like lesions

**Fig. 7:**
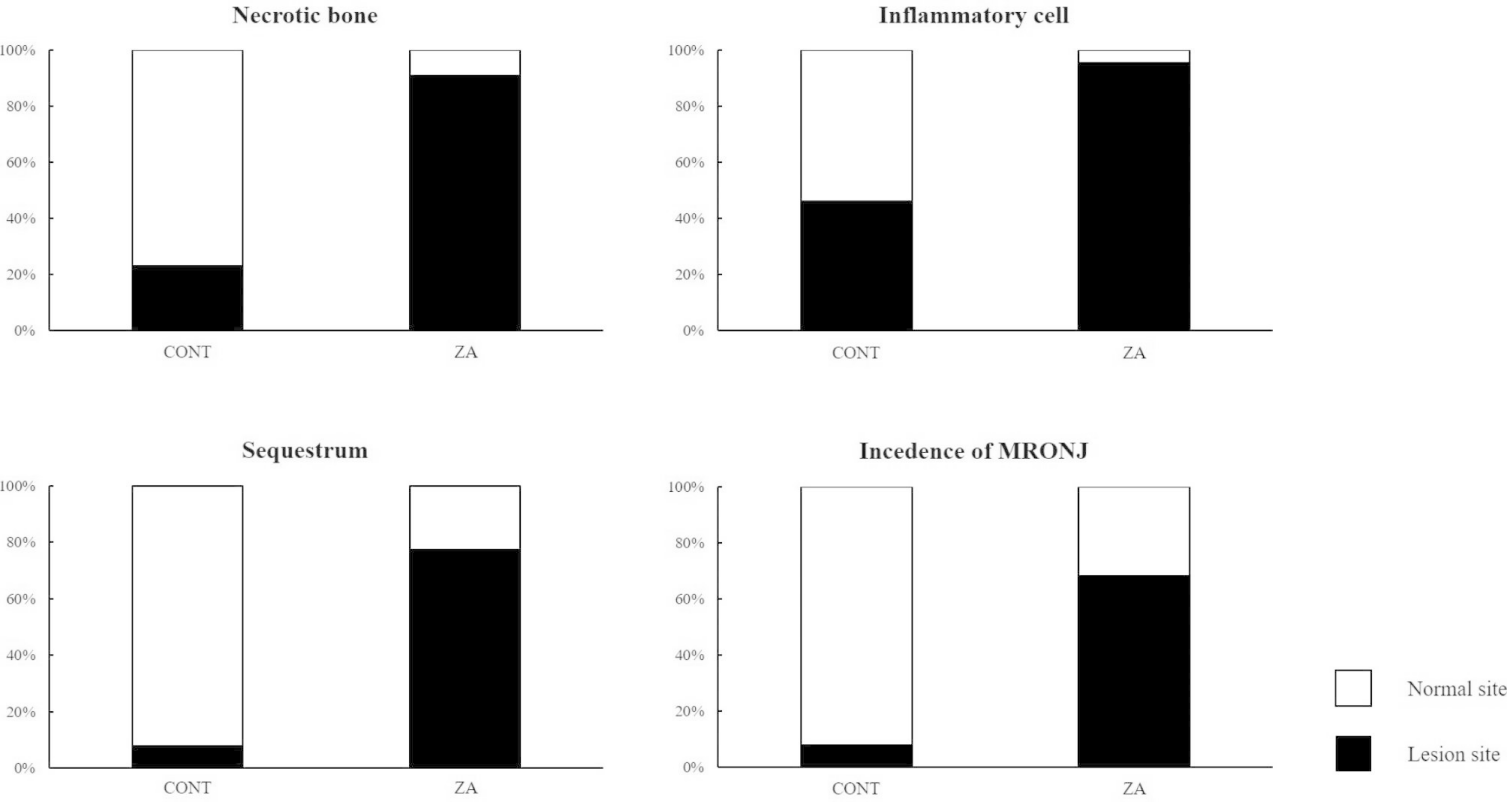
Histological evaluation of the incidence of lesions in the EXT socket *Abbreviations*: EXT = extraction.

MRONJ-like lesion were diagnosed based on the histological criteria (Fig. 7). The incidence of CONT group and ZA group were 68.1% (15 sites/22 sites) and 7.7% (2 sites/26 sites), respectively. The incidence of MRONJ lesions was significantly higher in the ZA group than in the CONT group (*P* < 0.000).

## Discussion

In this study, MRONJ incidence was investigated when ZA was administered and tooth extraction was performed after inducing periodontitis in OVX rats. Although ZA improved the osteoporosis induced in the long bones, it also induced MRONJ onset.

Periodontal disease and caries are among the most common causes of tooth extraction [34]. MRONJ occurs in patients after tooth extraction, and the teeth cannot be restored owing to periapical infection with advanced dental caries or periodontal disease [20, 35, 36]. Inducing periodontal lesions was attempted by placing tooth ligation on the cervical portion of the teeth for 8 weeks before extraction. Duarte et al. reported that bacterial species commonly observed in humans were found in the biofilm around the ligature 42 days after ligation in the rats [37]. Liu et al. reported that a 28-day ligature could cause significant loss in the trabecular pattern of the alveolar bone in rats [38]. Therefore, the tooth ligation was kept for 8 weeks in our study, and this duration was adequate to induce periodontal lesions. However, there was no visible gingival inflammation or tooth mobility observed 8 weeks after ligation, which was an unexpected outcome. Additionally, the amount of alveolar bone resorption was less than expected. This could be explained by the rapid metabolism in rats and their remarkable ability to regenerate. Many previous studies have attempted to overcome the limitations of tooth ligation used to induce periodontal disease in animal models in various ways [39, 40]. It is necessary to include experimental data using different methods of inducing periodontal disease [41–43].

In the micro-CT analysis, as seen in previous studies, osteoporosis was induced in the long bone by performing OVX, and improved bone quality was observed with ZA administration; however, the mandible showed different findings. When MRONJ occurs, the BV/TV decreases because bone loss increases due to bone resorption and sequestrum formation. Due to the inhibitory action of ZA on osteoclasts, bone resorption is suppressed, BS/BV is lowered, and BS/TV is increased. Thus, there was substantial inflammation in and around the tooth extraction sockets in the ZA group in our study. In the ZA group, the Tb.Th and Tb.N values were high, but the Tb.Sp was low. These values are used to evaluate the characteristics of trabecular bone in osteoporosis, which shows decreased BV/TV, Tb.Th, and Tb.N and increased Tb.Sp; therefore, they can be used to evaluate the effectiveness of osteoporosis drugs. However, it is considered that the bone in a state of increased inflammation due to MRONJ should not be interpreted as an evaluation criterion for non-inflammatory trabecular bone. Summarizing the results of the tibia and jaw, we found that the BV/TV, Tb.Th, and Tb.N were high and Tb.Sp was low in the non-inflammatory state (tibia), but the BV/TV showed contrasting findings in the inflamed state (jaw) (Figs. 3 and 4). This is because osteosclerosis of the trabecular bone occurs when osteomyelitis develops.

MRONJ was diagnosed according to the histological criteria (exposed bone, necrotic bone, inflammatory cell infiltration, or sequestrum) at the extraction site. This was because there were MRONJ lesions in some rat specimens that could not be clinically detected and were only observed in the microscopic analysis. Although the definition of MRONJ is based on the macroscopic appearance in the clinic, MRONJ was diagnosed based on the following histological criteria under experimental conditions: (1) presence of ulcerative lesions with exposed and necrotic bone and/or osteolysis [44]; (2) presence of pseudo-epitheliomatous-like hyperplasia of the epithelium accompanied by inflammatory infiltration [31]; and (3) presence of sequestrum and bacterial colonies [8, 45]. The incidence of MRONJ in the ZA group was significantly higher than that in the CONT group. Notably, the incidence of 7.7% in the CONT group was unexpected. These results could be attributed to the general condition and root morphology of the rats. The rats used in this experiment could be considered immunosuppressed because they represented postmenopausal women with osteoporosis. Therefore, the extraction sites of the CONT group may have also shown inflammatory findings. The other reason is the morphological characteristics of the rat molars. Rat molars have more divergent roots than those of humans, resulting in more chances of remnant root pieces, which may affect the healing of the extraction sites. Fractured root was confirmed in 2 animals in the CONT group and 1 animal in the ZA group. Fractured root was 1 per tooth in all animals.

Many studies have been conducted to elucidate the pathogenesis of MRONJ, and there are several reports on those related to periodontal and pulp diseases in rats [46–50]. Particularly, when a surgical procedure such as tooth extraction is performed in a state of chronic inflammation such as periodontitis, the incidence of MRONJ is high [46–49]. Although various hypotheses have been proposed regarding the pathogenesis of MRONJ, the mechanism related to macrophage activation is considered the most influential [46]. It has been reported that MRONJ develops when ZA is administered in a periodontitis-induced rodent model [28]. Moreover, if tooth extraction is performed in the presence of periodontitis, the incidence of MRONJ increases, and the incidence of MRONJ is reduced when tooth extraction is performed in a healthy state in which inflammation is removed by treating periodontitis [51]. Interleukin-17 cytokine was found to increase the M1/M2 macrophage ratio in the mucosa surrounding the MRONJ affected area, suggesting that the activation of M1 macrophages acts as a major risk factor for MRONJ [52].

A limitation of this study is that a comparison of the incidence of MRONJ between ligated and non-ligated teeth was not performed. To investigate whether periodontal disease-induced extraction is a risk factor for MRONJ, ligated and non-ligated teeth should be extracted simultaneously from one specimen and analyzed to compare the differences of the extraction sites. The ligation period and ZA dose should be further adjusted in future studies. Moreover, an experimental design reflecting the withdrawal period before and after the extraction to simulate the patient’s situation is warranted.

## Conclusion

In conclusion, our findings suggest that the pathological inflammatory state of the periodontal tissue after ZA administration is likely to induce MRONJ after tooth extraction.

## Data Availability

The datasets used and/or analyzed during the current study are available from the corresponding author on reasonable request.

## List of abbreviations

BMD: Bone mineral density
BP: Bisphosphonate
BRONJ: Bisphosphonate-related osteonecrosis of the jaw
BS/BV: Specific bone surface
BS/TV: Bone surface density
BS: Bone surface
BV: Bone volume
IV: Intravenously
M1: First molar
M2: Second molar
MRONJ: Medication-related osteonecrosis of the jaw
ONJ: Osteonecrosis of the jaw
OVX: Ovariectomy
ROI: Region of interest
SC: Subcutaneously
Tb.N: Trabecular number
Tb.Sp: Trabecular separation
Tb.Th: Trabecular thickness
TV: Total volume
ZA: Zoledronic acid
